# Dietary Supplements as Source of Unintentional Doping

**DOI:** 10.1155/2022/8387271

**Published:** 2022-04-22

**Authors:** Vanya Rangelov Kozhuharov, Kalin Ivanov, Stanislava Ivanova

**Affiliations:** Department of Pharmacognosy and Pharmaceutical Chemistry, Faculty of Pharmacy, Medical University-Plovdiv, 4002 Plovdiv, Bulgaria

## Abstract

**Background:**

The substances used in sport could be divided into two major groups: those banned by the World Anti-Doping Agency and those which are not. The prohibited list is extremely detailed and includes a wide variety of both medicinal and nonmedicinal substances. Professional athletes are exposed to intense physical overload every day. They follow a relevant food regime and take specific dietary supplements, which is essential for the better recovery between trainings and competitions. However, the use of “nonprohibited” dietary supplements (DS) is not always completely safe. One of the risks associated with the use of dietary supplements is the risk of unintended doping—originating from contaminated products. The presence of undeclared compounds in the composition of DS is a serious concern. The aim of this study is to evaluate the risk of unintentional doping.

**Materials and Methods:**

Literature search was done through PubMed, Science Direct, Google Scholar, and Web of Science. Studies investigating the presence of undeclared compounds, in dietary supplements, banned by WADA met the inclusion criteria. The last search was conducted in June 2021. The present review is based on a total of 50 studies, which investigated the presence of undeclared compounds in DS.

**Results:**

The total number of analyzed DS is 3132, 875 of which were found to contain undeclared substances. Most frequently found undeclared substances are sibutramine and anabolic-androgenic steroids.

**Conclusion:**

More than 28% of the analyzed dietary supplements pose a potential risk of unintentional doping. Athletes and their teams need to be aware of the issues associated with the use of DS. They should take great care before inclusion of DS in the supplementation regime.

## 1. Introduction

Obligatory drug testing was introduced by the International Olympic Games in 1968 Committee. Since then, numerous doping cases were reported. The presence of prohibited substances and/or their metabolites in athlete samples (blood or urine) is considered a serious violation of the Anti-Doping Rules, according to the World Anti-Doping Code (WADC). Athletes are responsible even when a doping compound enters their bodies without their knowing, because according to WADC, it is considered athletes' “personal duty” to ensure that no prohibited substance enters their bodies [1].

Violations of the Anti-Doping Rules include not only unintended or attempted use by an athlete of a prohibited substance but also the use of a prohibited method. There are three main categories of prohibited methods: manipulation of blood and blood components, chemical and physical manipulation, and gene and cell doping [1].

Doping is considered a serious sports crime, which may cause many negative effects, including loss of championship titles, bans from participation in competitions, compromised reputation, and poor health. Since doping is considered a premeditation behavior, engaging in this behavior is mainly attributed to the athlete's decision-making process and moral values or obligations [2].

In the last 2 decades, there were many cases of unintended doping because of the use of dietary supplements (DS) with bad quality. In the same time, the trend of using DS by professional athletes is constantly growing [3]. Doping control statistics from the Olympic Games in Sydney and Athens in 2000 and 2004 show that [4, 5] 78 percent and 75.7 percent of tested athletes consumed food supplements in the three days before testing, respectively. Several studies indicate that the use of DS by elite athletes varies from 69% to 94% [6, 7]. The DS are an important part of athletes diets and may provide some benefits like fast recovery after intense exercise regimens, better exercise performance, and enriching the diet [8]. The most commonly used categories DS by professional athletes are vitamins and minerals, proteins and amino acids, stimulants, weight loss DS, and others [7–9]. The majority of dietary supplement users are not aware that the administration of such materials can be hazardous. They believe that dietary supplements are approved from government agencies and that they are tested for safety and efficacy [10].

Unlike medicines, the dietary supplements are not tested for quality before releasing on the market. Legalization about these products is quite liberal all over the world. However, in Canada, dietary supplements are referred as natural health products (NHPs) and are considered as a specific category of drugs under a specific regulation of the Natural Health Products Regulations. Products and manufacturers must be licenced, and the dietary supplements must pose evidence of safety and efficacy [11]. In Europe and the US, there is no premarket safety requirements, and producers are responsible for safety of a dietary supplement [12]. In European Union, dietary supplements are regulated as foods under the Directive 2002/46/EC [13]. Legislation is mainly focused on vitamins and minerals. Regulation is mostly based on main integrities, and risk of adulterants is high [14]. There are no obligatory tests about the quality and quantity of the active compounds [15]. There are no obligatory tests about impurities, related compounds, and lead. In the US, dietary supplements are regulated by the US Food and Drug Administration (FDA) under the provision of the Dietary Supplement Health and Education Act (DSHEA) since 1994 [14]. Manufacturers of dietary supplements are not required to provide information's of efficacy or safety before they introduce supplements on the market. Manufacturers are free to establish their own practice guidelines, and FDA does not regulate quality, identity, or purity of the products [16]. DSHEA put FDA in a difficult position, with a requirement that FDA needs to prove that dietary supplements are adulterated rather than to require from manufacturers to prove a supplement safety. In other words, there are no premarket regulations. FDA only need to be notified if there are new integrities in the existing dietary supplement. Even more, FDA relies on information provided by manufacturers and public, which provide inadequate date of real picture [17, 18]. Even though FDA requires from manufacturers to report serious side effects, the level of those reports is low [19]. In 2011, 73% manufacturers inspected by FDA broke 1 or more regulations. Even if manufacturers adhere to the FDA's cGMP guidelines, this does not guarantee the absence of all contaminants [20]. GMP demands internal quality product testing and limited testing for adulterants [21]. There is no quality controls on active substances and label compliance. Dietary supplements are often adulterated and contaminated or fail to contain claimed active ingredient [12, 20]. Unfortunately, in the last few years, a lot of products with bad quality were introduced on the market [22–81]. Dietary supplement usage has progressively risen in various nations throughout the world over the years [82]. Global dietary supplement market was $191.1 billion in 2020, and there are predictions to reach $307.8 billion till 2028 [83]. Furthermore, a rising public awareness of the significance of nutrition in health maintenance, along with popular views that today's food is deficient in vitamins, is among the reasons that dietary supplement use is becoming a mainstream practice [84–86]. In the United States, more than half of individuals claim supplement usage, and in other studies, almost 40% have used dietary supplements in the previous 30 days [87, 88]. Despite the fact that dietary supplements are associated with beneficial health behavior, some DS can have negative consequences. In the United State, 23000 emergency visits are due to dietary supplement-related adverse events [89]. Dietary supplement use with no clear understanding by athletes may lead to use of stimulants unintentionally because they are unaware that food, drinks, supplements, or drugs may contain adulterants [90, 91]. In Europe and worldwide, there is no specific regulation established for sports supplements, and they are under existing regulations [92]. WADA list of prohibited substances is not considered in the current legalizations, and athletes are exposed like any other [93, 94]. Maintaining a good health by taking needed nutrients, correcting nutrient deficiencies, or supporting the immune system represents a real challenge. Even more, their carrier and reputation can be drastically damaged if adulterants exist on the WADA prohibited list [8]. It has been reported that some athletes have tested positive for doping due to use of dietary supplements that had poor labeling or contamination of the product [95]. Approximate 6.4% to 8.8% of reported doping cases are the result of undeclared substances in dietary supplements [96]. Therefore, one of WADA's (World Anti-Doping Agency) main antidoping strategies, in addition to doping control, is to enhance athletes' antidoping awareness [97].

Subsequent studies in recent years have found that many supplements contain undeclared compounds like prohormones or anabolic androgenic steroids (AAS) such as stanozolol, methandienone, boldenone, and oxandrolone [22, 23, 38–40, 44, 53, 61, 63, 74, 75, 80, 98]. The intake of such products will definitely lead to unintended doping. This problem is an extremely serious, and its possible solutions must be considered.

According to data published by the World Anti-Doping Agency, 44% of positive doping cases include anabolic steroids. This category includes endogenous AAS such as testosterone and nandrolone and many other anabolic agents such as selective androgen receptor modulators (SARMs) and clenbuterol. The first cases of AAS contamination in dietary supplements were reported in 2000 with norandrosterone [24].

The purpose of this paper is to review and assess the risk of inadvertent doping due to the use of dietary supplements and to pay particular attention to the ways of preventing this type of doping.

## 2. Materials and Methods

This is a descriptive study, consisting of a bibliographic review. In the first phase of our study a detailed search strategy was processed. To find eligible studies, we searched the following scientific electronic databases: PubMed, Google Scholar, Science Direct, and Web of Science, with no language restrictions. The search was undertaken using the following key words in PubMed, and it was adapted to other scientific electronic databases: (“unintentional doping” OR “inadvertent doping” OR “doping in sports”) AND (“food supplements” OR “dietary supplements” OR “nutritional supplements”) AND (“prohibited substances” OR “banned substances in sport” OR “undeclared substances”).

Other relevant sources were found by looking through the references of related articles. No additional filters were added, and the last search was conducted in June 2021. The Zotero program was used to generate the references in this paper. All possible relevant full texts were independently selected by 2 authors. Disagreements were resolved through discussion. In the case of an objection to the judgment, the third author was involved.

**Figure 1:**
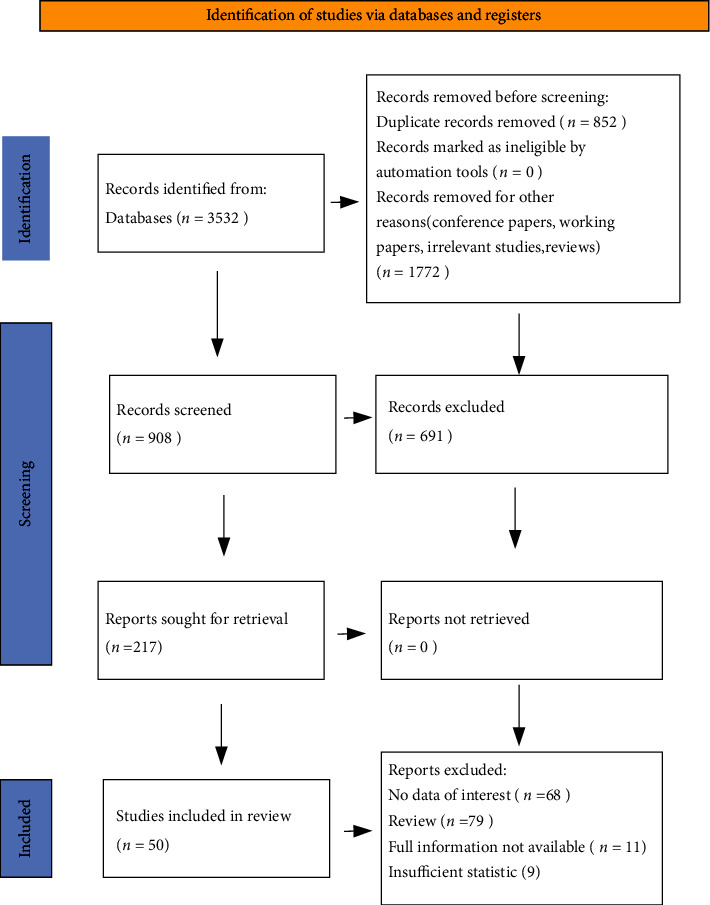
PRISMA 2020 flow diagram for new systematic reviews which included searches of databases and registers only.

In the second phase, we followed the PRISMA (Preferred Reporting Items for Systematic Review and Meta-Analysis) presented in Figure 1 [99], as a guide for a systematic review. Systematic reviews frequently reveal a lack of understanding of general guidelines that make them repeatable and scientific. PRISMA provides an expert-approved standard method that uses the guide checklist strictly followed in this article to help ensure the quality and repeatability of the revision process [100, 101].

In the third phase, studies were chosen based on exclusion and inclusion criteria. The exclusion criteria were articles written in a language other than English or German, articles involving topics that were clearly irrelevant (they do not pose results in field of interest), animal studies, and human studies. We did not include studies that were only concerned with intentional doping. To ensure reliability and quality, working paper, conference paper, and reviews are excluded. Inclusion criteria were as follows: only original research studies were included, but secondary research studies were subject to a screening process; publication in a peer-reviewed journal; and studies examining the presence of substances, metabolites, and markers banned by WADA in dietary supplements [93].

In the fourth phase, the chosen articles were read fully and extra articles identified from their references were also reviewed.

One researcher read the complete text of the preselected articles to apply eligibility criteria, while a second researcher double-checked the selections to ensure that all studies were included. To avoid missing information, one author obtained data and had it confirmed by another author. The extracted data set contains number of analyzed dietary supplements, number of dietary supplements that contain undeclared substances, undeclared substances which are banned by WADA, methods for detection of undeclared substances, and country of origin [93].

## 3. Results and Discussion

A total of 50 manuscripts were selected and included in the present bibliographic review. Data was summarized in Table 1.

Table 1 presents information on studies performed on dietary supplements containing substances listed in the WADA Prohibited List. Data from 50 research articles were processed. The articles included in the table are published in the period between 1996 and 2021. The total number of studied dietary supplements in these articles reaches 3132, of which 875 show the content of undeclared substances. The most common is sibutramine found in a total of 14 of 50 articles. 248 from 875 dietary supplements contain sibutramine (28.34%), testosterone and other anabolic steroids in 228 (26.06%), 1,3-dimethylamylamine (DMAA) in 58 (6.62%), fluoxetine in 192 (21.37%), and higenamine in 15 of the 875 dietary supplements (1.71%). Various diuretics and SARMs have also been identified as undeclared substances in dietary supplements.

The methodology most used for the detection of undeclared substances was gas chromatography combined with mass spectrometry (GC-MS), in seven research articles, followed by liquid chromatography combined with tandem mass spectrometry (LC-MS/MS), in seven research articles; nuclear magnetic resonance (NMR) in 4; ultra-high-performance liquid chromatography tandem mass spectrometry (UHPLC-MS/MS) in 3; GC-MS combined with high-performance liquid chromatography with a diode-array detector (HPLC-DAD); liquid chromatography electrospray ionization tandem mass spectrometry (LC-ESI-MS/MS); and LC-MS in 2.

We found that almost 28% of the studied dietary supplements pose a potential risk of unintentional doping. Doping substances are not specified as components in the dietary composition claimed on the labeling, or amounts stated differ from their real content [23–81]. The studies were performed mainly in Europe. Some of the products were purchased via the Internet and others from local shops and pharmacies. Contaminated products originated all over the world, mostly from the USA, the Netherlands, the UK, Italy, and Germany; some of the samples originated from China and Southeast Asian countries. No matter where a consumer lives, there is a chance to buy a contaminated DS. The results indicate that a large amount of dietary supplements contain undeclared substances, which with consistent growing of supplementation use from athletes represent a high risk [23–81]. In 2004, Geyer and team published a study where 634 dietary supplements were analyzed from 15 different countries, 14.8% positive supplements showed anabolic steroids concentrations from 0.01 *μ*g/g up to 190 *μ*g/g [36]. An additional administration study demonstrated that use of supplements containing nandrolone prohormones at a complete amount of 1 *μ*g can reach levels higher than the WADA minimum required performance level (MRPL) of 2 ng/mL [102]. Researchers Baume et al. performed a very important study on the purity of dietary supplements purchased from different websites: 103 dietary supplements, from the following categories: stimulants, amino acids, creatine, and others. It was established that 18% of the analyzed dietary supplements contained undeclared anabolic steroids or their precursors [37]. High amount of metandienone was found in three supplements in a range from 0.41 mg/g up to 17.30 mg/g, which would increase levels more than urinary MRPL of 2 ng/mL [101]. This review indicates that anabolic steroids are present in 26.06%, supplements that show content of undeclared substances, in various concentrations. Use of some of these products will lead not only to antidoping rule violations but also to some negative health consequences [103–106]. Anabolic steroids may cause the body's natural hormone production to be disrupted. This might cause both reversible (infertility) and irreversible changes in the organism like gynecomastia [103, 107, 108]. The intake of anabolic steroids is also associated with cardiovascular side effects like hypertension, left ventricular hypertrophy, impaired diastolic filling, and thrombosis. Other serious side effect related to steroid admission is hepatotoxicity [103]. Nonbanned medications do not provide an enhancement in athletic performance, but drug-drug interactions (DDIs) can occur when two or more pharmacologically active chemicals are administered together, particularly in the case of unintentional use of doping substances. Anabolic steroids may interact with anticoagulants (such as warfarin), increase their effect, and raise the risk of bleeding complications [109–112].

The adverse effects of anabolic steroids are correlated with the prolongation of the intake. Usually, dietary supplements are taken for a long period of time several times per day that may expose the consumers to unexpected use of steroids and serious side effects. Prolonged use can potentially lead to cardiomegaly, and they are also linked to sudden cardiac death [113, 114]. Cases of liver carcinoma were related to long use of anabolic steroids [115, 116].

Another commonly detected undeclared compound in dietary supplements is sibutramine. It is mainly detected in weight-loss dietary supplements [31–34]. Sibutramine which is a reuptake inhibitor of serotonin and norepinephrine (SNRI) was approved in 1997 for treatment of obesity but was banned in 2010 because of the risks from cardiovascular disorders. In the past, the recommended dose was 10 mg once daily, with modification up to 15 mg [31]. The main reason sibutramine is not more used as a medicine is the higher number of cardiovascular events which were observed in people taking sibutramine containing drugs: hypertension, tachycardia, arrhythmias, and myocardial infarction [117–120]. In 2014, Kim and team analyzed 188 dietary supplements and found that 29 contained sibutramine in concentrations 0.03–132.40 mg/g [32]. In 2016, Adela Krivohlavek and team reported a similar worrying trend regarding dietary supplements available in Croatia. Of the 123 supplements analyzed, sibutramine was detected in 20%. The highest content was 26.41 mg/g [33]. According to our findings, sibutramine is the most commonly detected undeclared substance. Sibutramine is not a safe compound; its presence in food supplements poses serious health risks both for athletes and other individuals. Consumers who have unintended intake of sibutramine because of a contaminated dietary supplement may feel increases in the blood pressure, arrhythmias, or other side effects like dry mouth, trouble sleeping, headache, flushing, or joint/muscle pain [117, 118, 120, 121]. If these consumers are professional athletes, the intake of sibutramine will result also in positive doping cases. However, most of the customers of DS consider these products safe, without side effects and contraindications. Customers do not expect the presence of undeclared compounds in DS. For consumers with cardiovascular disease, the intake of products containing undeclared sibutramine could cause serious consequence [15]. Moreover, serious drug interactions are associated with sibutramine intake.

Sumatriptan and selective serotonin reuptake inhibitors (SSRIs), for example, should not be used with sibutramine because serotonin syndrome can occur. Use of monoamine oxidase inhibitors (MAOIs) and sibutramine can lead to increase of blood pressure [122, 123]. The unintentional intake of compound like sibutramine because of contaminated DS could affect overall health, the intake of medicines which are used for management of serious diseases, and a professional sport career [124–126].

In 2008 selective androgen receptor modulators (SARMs) were added to WADA's banned list [1]. Since the first analytical finding for SARM andarine in 2010, the number of this class of compound has been steadily increasing. The most common source of androgen receptor modulators is dietary supplements [25]. In 2018, a number of these compounds were reported as AAFs, including ostarine (currently the most common SARM) and ibutamoren. In 2020, Leaney et al. performed a study for detection of androgen receptor modulators in dietary supplements. Their team analyzed 20 dietary supplements. Only six of the analyzed samples were in accordance with the labeling [26]. In the other samples, the following were detected: andarine, ostarine, ibutamoren, and arimistane. Amounts of these undeclared compounds were measured, in a range from approximately 0.6 mg/capsule to 6.8 mg/per capsule. Use of SARMs is associated with liver toxicity and higher risk of myocardial infarction and stroke [127].

Stimulants are a class of compounds which stimulate CNS activity. They are one of the most established doping agents. The most common methods for detecting stimulants are GC-MS and LC-MS. The MRPL for stimulants is set at 100 ng/mL, except octopamine, for nonthreshold substances [101]. Although detection methods are sensitive, a large number of athletes still resort to stimulants. Stimulants have also been identified in a number of dietary supplements, most commonly as intentional impurities products designed for weight loss for quick results [27]. In addition, naturally occurring stimulants in plants can be tricky for athletes because the content varies between species and different names of substances and plants.

The stimulant commonly found in weight loss products is 1,3-dimethylamylamine (DMAA) [28, 29, 51]. Concentrations of DMAA in those study are varied from 1 mg/g up to 415 mg/g. In our review, 58 dietary supplements contain DMAA. The drug is a natural component in geranium plants, and it is used as a nasal decongestant. Use of DMAA might increase blood pressure, tachycardia, and risk of heart attack or stroke [128, 129]. This is especially critical for people who are hypertensive or who use other supplements or medications that are known to raise blood pressure.

Higenamine has been on the World Anti-Doping Agency's (WADA) prohibited list as a 2-agonist that is prohibited at all times for athletes since 2017 [1]. Cohen and his colleagues discovered higenamine in 24 easily available supplements, the majority of which were marketed for weight loss and energy enhancement. Doses were up to 62 ± 6.0 mg per serving [43]. WADA established criteria for higenamine as a prohibited substance, stating that analytical results of less than 10.0 ng/mg should not be reported. Our findings show that higenamine was found in 15 dietary supplements, in which concentrations were in a range from 0.018 mg up to 62 mg. Even though some amounts are found to be very low per serving, long-term use could potentially lead to unintentional doping. The intake of those products can increase blood pressure and cause irregular heartbeats [130, 131]. In human clinical studies, slight and transient side effects such as dizziness, nausea, heart palpitations, and dry mouth have been reported [131–133]. Higenamine may reduce the effectiveness of blood pressure medications and blood thinners [134, 135].

In essence, accidental doping can be avoided and every effort must be made to prevent unintended doping cases. Study on awareness of unintentional doping among athletes showed that only 40.6% refused to eat an unfamiliar food that was given to them, and only 16.1% read the ingredients list before consumption [136]. The best prevention is better analytical control, education, and information. WADA established ADEL (Anti-Doping Education and Learning platform), an online platform that helps in education and supports the antidoping community. [137]. Athletes and their teams need to obtain information about dietary supplements and to ensure that labeled substances are not on the WADA prohibited list [93]. Other agencies, such as the Court of Arbitration for Sport (TAS) [138], inform athletes of registered doping cases and provide information of possible banned substances in different sources. Organizations such as the US Anti-Doping Agency (USADA) [139] and the UK Anti-Doping Authority (UKAD) [140] also offers information about prohibited drugs. Furthermore, there are websites which evaluate the safety of dietary supplements such as Informed Sports in the UK [141] or the Cologne List in Germany [142]. One of the most easy accessible ways to check dietary supplements for banned substances is applications. NSF International launched a NSF Certified for Sport® application, which provides an information of tested and safe products [143]. Medi-Check Global DRO is based on the WADA prohibited list and contains the information about prohibited medications in Switzerland, Canada, the United Kingdom, and the USA [144]. The UK Anti-Doping application Clean Sport provides up-to-date antidoping information [145]. Sport Integrity Australia helps athletes to find low-risk supplements, also possibility to check medications, and to fulfill educational course [146]. It is recommended to check whether a product contains prohibited substances on various places dedicated to assessing the purity of dietary supplements. Purchasing dietary supplements from pharmacy or a major health store provides some assurance of better quality that those from the Internet or private individuals. If labeling does not provide information of content, dietary supplement should be avoided [36]. Again, unintentional doping can occur from lack of labeling or cross-contamination.

Current strategies for production and control of dietary supplements are not efficient to ensure safe products [147]. The lack of analytical control of dietary supplements before they are introduced to the market, as well as the use of poor manufacturing practices, has led to the availability of many unsafe products [148]. GMP relays on validated analytical methods and standard reference materials, and for a lot of dietary supplements, they are not available [149, 150]. Dietary supplement industry is growing rapidly. In Canada, till 2011, 43000 product licences were allowed [14]. More than 86000 new dietary supplements were introduced to the market in the period from 1994 to 2014 only in the US [20]. The safety data was received for only 250 new integrities [151]. Because of the low premarket investment, dietary supplements are getting easily to the market. In 2013, from 14 995 dietary supplement manufacturers, FDA inspected only 416 [20]. The agency does not have adequate system of control. Even officially banned substances like sibutramine and Ephedra sinica are used from manufacturers to provide “effective” supplements, which are threatening public health [12]. A study by Cohen at el. revealed that 67% of 27 tested dietary supplements banned from the FDA in the period from 2009 to 2012 were available on the market in 2014 [152]. DNA-based methods indicate that 27% of 5957 herbal products, from 37 countries, had adulterants and did not have label compliance [153]. New analytical methods cannot catch up to numerous integrities in dietary supplements. Urgent legalization changes are needed to ensure safe and good quality products. Premarket regulations on safety and efficacy are necessary. However, this is a long and а slow process, which cannot happen simultaneously globally. Nowadays, only several countries have strong demands about dietary supplements (example: Canada and Australia) [14, 154]. If the legislation about dietary supplements does not change, many new cases of unintended doping will appear in the next years. Moreover, such kind of products represents a serious health risk both for professional athletes and other consumers. In our view, the teams of professional athletes should analyzed all dietary supplements in accredited laboratories before including this product in the regime of the respective athlete. The analytical control is the best strategy for prevention of unintended doping. Our recommendation is that before inclusion of any DS in the regime of a professional athlete, an analytical control should be performed. This analytical control should be done only in accredited laboratories. It should establish not only the quality of the products (identification and quantification of the main ingredients of the DS) but it should also provide information about the presence of undeclared compounds, in which intake could result in positive doping cases. The detection of undeclared AAS, sibutramine, or other doping compounds could be reliably performed using techniques like GC/MS or HPLC/MS.

Limitations of this work can consider the fact that not all articles which investigate the presence of prohibited substances analyzed substances with same methodology and they analyzed different integrities, which open possibility of presence of more prohibited substances in revealed dietary supplements.

## 4. Conclusions

Almost 100% of athletes take DS because this ensures fast recovery, good performance, and enriched diet. In the same time, recent studies reported that many dietary supplements contain undeclared substances like sibutramine, anabolic steroids, hygenamine, and 1,3-dimethylamylamine and athletes are in a risk of inadvertent doping. This is a serious prerequisite for professional athletes to become victims of unintentional doping, taking without suspecting “prohibited substances.” The presence of unsafe dietary supplements is a result of the liberal regulation of these products around the world. A key element that is missing in the regulation of dietary supplements is the mandatory analytical control to ensure accurate quality and quantity of active substances and the absence of impurities. The intake of contaminated DS carries not only a risk of positive doping tests but also a risk to the health of the consumers. About 28% of the supplements analyzed in our study show the presence of undeclared substances. 28% is the risk of unintentional doping if a professional athlete includes untested dietary supplements in his/her diet. We consider that there is a very strong relationship between athletes' knowledge about doping and its prevention. A key point in doping prevention is the proper education, which provides enough information about doping. Another key point is the careful choice of the dietary supplements. Athletes and their teams must collect information on dietary supplements and check that the compounds listed are not on the WADA prohibited list. Moreover, because of the risk of presence of undeclared compounds, our recommendation is that the teams of the professional athletes who are responsible for their preparation should analyze all dietary supplements in accredited laboratories before including them in the regime of the respective athlete. Urgent legalization changes are needed to ensure safe and good quality products.

## Figures and Tables

**Table 1:** Information of studies showing proof of analytical analysis of undeclared substances banned by WADA in dietary supplements.

Number of analyzed dietary supplements	Number of dietary supplements that contain undeclared substances	Undeclared substances	Main results	Methods for detection	Country of origin	Ref.
634	94	Теstosterone, 4-norendionon, 4-аdendion, 5-adendiol, nandrolone, boldenone, dehydroepiandrosterone (DHEA)	14.8% positive supplements showed anabolic steroid concentrations from 0.01 *μ*g/g up to 190 *μ*g/g	GC-MS	Germany	[36]
75	7	DHEA, 19-noranrostenedione, ephedrine	Seven products had hormones that were not listed on the labels, and two more products contained ephedrine and caffeine that were not clearly labeled	GC-MS	Switzerland	[23]
103	3	Nandrolone, testosterone, 4-androstenediol, androstenediol, androstenedione, bolasterone, boldenone, clostebol, DHEA, dihydrotestosterone (DHT), drostanolone, fluoxymesterone, mesterolone,4-norandrostenediol, 5-androstenedione, 5-norandrostenediol, 19-noranrostenedione, metandienone, metenolone, methyltestosterone, norethandrolone, oxandrolone, oxymesterone, stanozol, oxymetholone, testosterone propionate, 5-norandrostenedione	An amount of metandienone in three supplements in a range from 0.41 mg/g up to 17.30 mg/g was found	GC-MS	Switzerland	[37]
12	11	Testosterone, 5-androstenediol, 4-androstene-3,17-dione, 5-androstene-3,17-diol, 19-androstene-3b, 17b-diol	Supplements did not meet the labeling requirements. One supplement contained 10 mg of testosterone; another contained 77% more than the label stated; 11 of the 12 contained less than what was indicated	HPLC, GC-MS	USA	[38]
64	8	Nandrolone decanoate, testosterone decanoate, 4-androstendion, ephedrine, DHEA	Concentration range: 1–25 ng/g	LC-MS/MS GC-MS	Italy	[39]
19	15	Тestosterone, beta-boldenone, alfa-boldenone, beta-nortestosterone	Concentrations between 0.01 mg/g and 2.5 mg/per capsule or tablet were found. Testosterone was in most samples (50%)	LC-MS/MS ESI	Belgium	[40]
16	9	1,3-Dimethylamylamine (DMAA)	Concentration range: 3.1 mg/g-415 mg/g	NMR	Germany	[28]
112	6	Dehydroepiandrosterone (DHEA)	Concentration range:1-500 ng/g	Androgen bioassay	New Zealand	[41]
19	2	Higenamine	Concentrations 6.42 ng/mL and 18.93 ng/mg were found in two samples	UHPLC-MS/MS	Serbia	[42]
24	5	Higenamine	Dosages of up to 62 ± 6.0 mg per serving were found	UHPLC-MS/MS	USA, Netherlands	[43]
6	6	D6-Methyltestosterone, methasterone, prostanozol	After positive doping samples, 6 dietary supplements were tested and all have undeclared substances	GC-MS	Germany	[44]
10	2	Sibutramine	More than 20 mg per tablet was found	Ultra-high-performance liquid chromatography-high-resolution mass spectrometry (UHPLC-HRMS) UHPLC-MS/MS	Bulgaria	[31]
20	14	Andarine, ostarine, ibutamoren, arimistane	The amounts of these undeclared analytes ranged from 0.6 mg per capsule to 6.8 mg per capsule	UHPLC LC-HRAM (high-resolution accurate mass)	UK	[26]
188	29	Sibutramine	Concentrations of detected sample range from 0.03 mg/g up to 132.40 mg/g	LC-MS/MS	South Korea	[32]
123	24	Sibutramine	Sibutramine contents in green coffee samples were 0.014–2 438 mg/kg; the highest content of sibutramine was found in one capsule sample (26 410 mg/kg)	LC-ESI-MS/MS	Croatia	[33]
200	27	Synephrine, yohimbine, 5-hydroxytryptophan, DHEA	The concentrations of detected samples ranged from 0.51 mg/g up to 226 mg/g	LC-MS/MS	South Korea	[35]
13	13	DMAA	Concentration range from 0.1 mg/g to 110 mg/g	HPLC-MS	USA	[29]
8	7	Sibutramine, phenolphthalein, bumetanide, phenytoin	Concentrations of sibutramine ranged from 6 mg up to 57 mg per capsule	LC-MS GC-MS	Iran	[34]
50	24	Sibutramine, rimonabant, desmethylsibutramine, didesmethylsibutramine, sildenafil	Concentrations of sibutramine ranged from 0.1 mg up to 21 mg per capsule	HPLC-DAD-MS/MS	Netherlands	[45]
52	11	Theobromine, theophylline, pseudoephedrine, caffeine, hydrochlorothiazide, yohimbinе	Hydrochlorothiazide content found: 0.01 mg-0.37 mg Pseudoephedrine content found: 0.03 mg-11 mg	UHPLC-DAD	Saudi Arabia	[48]
5	5	Sibutramine, 4-hydroxyamphetamine, caffeine, theophylline	Sibutramine content found: 15 and 26 mg/g Theophylline content found: 0.2-0.3 mg/g	HRAM screening LC-MS/MS	Italy	[49]
Dietary supplement Dexaprine		Synephrine, oxilofrine, deterenol, yohimbine, caffeine, theophylline	Oxilofrine content found: 1-5 mg	Ultra-high-performance liquid chromatography-quadrupole time-of-flight mass spectrometry (UHPLC-QTOF-MS)	Netherlands	[50]
27	18	Demelverine, hordenine, N, N-dimethyl-phenethylamine, synephrine, N-methyl-*β*-phenethylamine, methylsynephrine	Unlisted DMAA was identified in three products with concentrations ranging from 18 to 120.9 mg/daily dose, while unlisted DMBA was found in one product with a concentration of 108 mg/daily dose	LC-QTOF-MS	USA	[51]
30	11	4-Androstenedione	4-Androstenedione was found in a range of 1.04 ng/g-2.52 ng/g	UPLC-MS/MS	Iran	[53]
108	53	1,3-Dimethylamylamine, sibutramine, methylphenidate, synephrine	DMAA was present in 20% of the dietary supplements. Out of the 108 samples, almost 50% were positive for sibutramine and 10% for methylphenidate	DART-MS/MS	Brazil, USA	[54]
11	3	Sibutramine	Concentrations of sibutramine ranged from 3.5 mg/g to 4.08 mg/g	Electrochemiluminescence (ECL)	China	[55]
16	13	Benfluorex, fluoxetine, pseudoephedrine, tiratricol	Pseudoephedrine 2.6-55.56 mg Fluoxetine 0.14-7.53 Benfluorex 0.01-0.07 Tiratricol 0.01-0.67	LC-UV	Italy	[57]
120	29	Sibutramine, fluoxertine	Sibutramine 0.26-113.22 mg per capsule Fluoxetine 1.80-101.08 mg per capsule	UHLPC-LTQ-Orbitrap MS	China	[58]
17	8	1,3-Dimethylamylamine (1,3-DMAA), 1,4-dimethylamylamine (1,4-DMAA), 1,3-dimethylbutylamine (1,3-DMBA), higenamine, deterenol, phenpromethamine, oxilofrine, octodrine, beta-methylphenylethylamine	Concentration range for detected stimulants were 2.7-17 mg of deterenol, 1.3-20 mg of phenpromethamine, 5.7-92 mg of beta-methylphenylethylamine, 18-73 mg of octodrine, 48 mg of higenamine, 18-55 mg of oxilofrine, 17 mg of 1,3-dimethylamylamine, 1.8 mg to 6.6 of 1,3-dimethylbutylamine, and 5.3 mg of 1,4-dimethylamylamine	UHPLC quadrupole-Orbitrap MS	Netherlands	[59]
32	16	Phenethylamine, synephrine, oxilofrine, hordenine, beta-methylphenethylamine, N-methyltyramine, octopamine, deterenol	High concentrations of phenethylamine were found in two products (29.9 and 26.8%, respectively). Synephrine was detected in 15 products with various contents ranging from 0.5 to 5.7%. The concentration of oxilofrine in these products ranged from 0.2 to 11.6%; deterenol with a content of 4.1%. The potential intake of the compound was estimated to be 28.7 mg	NMR	USA	[60]
40	32	Тestosterone propionate, testosterone phenylpropionate, testosterone isocaproate, testosterone decanoate, testosterone cypionate, testosterone undecanoate, stanozolol, drostanolone propionate, trenbolone acetate, oxymetholone, methandrostenolone	Eight of the 40 samples analyzed lacked all or some of the components listed on the label	NMR	Brazil	[61]
160	89	Sibutramine, fluoxetine, phenolphthalein, orlistat	Sibutramine was detected in 26% of the capsules, with sibutramine concentration ranging from 0.1 to 22 mg per dosage unit (capsule). The dosages found in this study varied from 0.05 to 56 mg per capsule, with phenolphthalein 0.8 mg-29 mg per capsule fluoxetine	NMR	France	[62]
48	3	Nandrolone, testosterone, dehydroepiandrosterone (DHEA), 5*α*-androstan-3,17-dione, 19-norandrostendione, progesterone	Positive tests included substances at values ranging from 0.022 to 0.398 mg/kg	Two-dimensional gas chromatography coupled to time-of-flight mass spectrometry (GC×GC–TOF-MS)	Czech Republic	[63]
20	5	Phenethylamine, N, N-diethyl phenethylamine, N-ethyl-*α*-ethyl-phenethylamine	N-Ethyl-*α*-ethyl-phenethylamine was found in a range of 6.48-23.4 mg per capsule Phenethylamine in a range of 1.39-24.7 mg	LC-MS-MS	USA	[64]
9	6	Ephedrine	Ephedrine range: 1.08-3.54 mg per capsule	HPLC	USA	[65]
7	7	1,3-Dimethylamylamine	DMAA range from 1.1 mg/g to 32 mg/g was found	UPLC-MS/MS	USA	[66]
2	2	Sibutramine, phenolphthalein	24.71 mg sibutramine and 48.20 mg phenolphthalein were found	HPLC	Romania	[67]
10	3	Sibutramine, fluoxetine	All three adulterate supplements were manufactured in China and labeled as food supplements containing only plants—“100% natural products”	Fourier-transform infrared spectroscopy (FTIR) GC-MS	Romania	[68]
20	3	Ephedrine, caffeine	Ephedrine range: 31–90 mg/dose	HLPC	Romania	[69]
26	18	Sildenafil, tadalafil, vardenafil	69% of the analyzed products that are found on the Romanian market turned out to be adulterated	LC-MS	Romania	[70]
80	76	Diphenoxylate, tramadol, codeine, sertraline, fluoxetine	Diphenoxylate and tramadol are found at concentrations of 1.4–4 mg/capsule and 67–150 mg/capsule, respectively	GC-MS	Iran	[72]
138	74	Fluoxetine	In comparison to supplements produced and purchased in South Africa (5194 ng/g), supplements imported and purchased in South Africa showed a greater amount of fluoxetine contamination (20052 ng/g)	LC-MS	South Africa	[73]
24	16	13 anabolic steroids	Amount of found substances per capsule: DHEA: 16 mg-28 mg Androstenedione: 1 mg-9 mg Methasterone: 2.4 mg-8 mg Testosterone: 7 mg Furazabol: 31 mg Androst-4-ene-3,11,17-trione: 61 mg	GC-MS HPLC DAD UV-VIS NMR	UK	[74]
198	5	Testosterone, stanozolol, 5*α*-hydroxylaxogenin	According to the detected concentration and daily serving size, it was found that 0.34 *μ*g of stanozolol would be consumed per day.	LC-MS/MS	Korea	[75]
36	8	Ephedrine, pseudoephedrine	Ephedrine at a concentration ranging from 370 ng/g to 1000 ng/g (in one case, ephedrine was not declared in the label) pseudoephedrine in 540 ng/g	LC–HRMS	Italy	[76]
52	26	Sibutramine	Half of the supplements included sibutramine in levels as high as 35 mg per capsule	HPLC-UV	Switzerland	[77]
124	6	Diuretics	Nearly 5% of the samples were found to be contaminated with diuretics at values ranging from 0.051 to 162 mg/g	UHPLC-Q-Orbitrap	Republic of Korea	[78]
3	3	Sibutramine	A new method was developed for the identification and quantification of the sibutramine by HPTLC	HPLC HPTLC	Turkey	[79]
18	8	4-Androstene-3*β*,17*β*-diol, 5*α*-androstane-3*β*,17*β*-diol	Concentration that was around or above 0.01 mg per capsule or tablet was found	LC-MS/MS	Netherlands	[80]
2	2	Мetandienone, Stanozolol	Metandienone with a concentration of 16.8 mg/tablet and stanozolol 14.5 mg/tablet were identified. Norandrostenedione was also found in both products, along with small levels of numerous additional steroids	GC-MS HPLC-DAD	Germany	[81]
